# Whole Slide Imaging and Its Applications to Histopathological Studies of Liver Disorders

**DOI:** 10.3389/fmed.2019.00310

**Published:** 2020-01-08

**Authors:** Rossana C. N. Melo, Maximilian W. D. Raas, Cinthia Palazzi, Vitor H. Neves, Kássia K. Malta, Thiago P. Silva

**Affiliations:** ^1^Laboratory of Cellular Biology, Department of Biology, Federal University of Juiz de Fora, Juiz de Fora, Brazil; ^2^Faculty of Medical Sciences, Radboud University, Nijmegen, Netherlands

**Keywords:** whole slide imaging, digital pathology, digital slide, virtual microscopy, histopathology, liver disorders, histology, hepatic tissue

## Abstract

Histological analysis of hepatic tissue specimens is essential for evaluating the pathology of several liver disorders such as chronic liver diseases, hepatocellular carcinomas, liver steatosis, and infectious liver diseases. Manual examination of histological slides on the microscope is a classically used method to study these disorders. However, it is considered time-consuming, limited, and associated with intra- and inter-observer variability. Emerging technologies such as whole slide imaging (WSI), also termed virtual microscopy, have increasingly been used to improve the assessment of histological features with applications in both clinical and research laboratories. WSI enables the acquisition of the tissue morphology/pathology from glass slides and translates it into a digital form comparable to a conventional microscope, but with several advantages such as easy image accessibility and storage, portability, sharing, annotation, qualitative and quantitative image analysis, and use for educational purposes. WSI-generated images simultaneously provide high resolution and a wide field of observation that can cover the entire section, extending any single field of view. In this review, we summarize current knowledge on the application of WSI to histopathological analyses of liver disorders as well as to understand liver biology. We address how WSI may improve the assessment and quantification of multiple histological parameters in the liver, and help diagnose several hepatic conditions with important clinical implications. The WSI technical limitations are also discussed.

## Introduction

Histological evaluation of hepatic tissue specimens is central not only for diagnosis and grading of numerous liver diseases in the clinical practice and research but also to understand different aspects of the liver biology (1–3). Distinctive hepatic microscopic features are important to narrow differential diagnosis, to establish disease extent and to guide therapeutic decisions. However, while manual examination of histological slides on the microscope classically provides diagnosis of different liver disorders, it is also considered time-consuming and limited for quantification of histological parameters, since only a small fraction of information is being harvested from tissue slides (4). Thus, conventional histopathological evaluations of tissue sections remain semi-quantitative with potential limitations in accuracy and objectivity (5).

The fields of digital pathology and histologic image analysis have grown considerably in the last decade in an attempt to improve the traditional histologic assessment in clinics and laboratories (6–9). Digital pathology refers to the processes of capturing, storing and interpreting of pathological specimens using images in digital file formats (10). There are assorted technologies that have been developed under the umbrella of digital pathology, including whole slide imaging (WSI), a technique that involves digitalization of entire histologic sections with the use of a digital slide scanner thus generating “digital slides” (10–12). High-resolution and wide-field microscopy images can be rapidly captured with WSI consequently offering highly detailed information about the tissue morphology. WSI associated with computational technologies also enables quantification of multiple histological parameters which provide insights into the disease pathogenesis and tissue/organ biology (10).

This review addresses the current status of WSI applied to the study of the liver in different disease contexts. Moreover, the applications of WSI to understand multiple features of liver biology as well as its potential strengths and technical limitations are addressed.

## Whole Slide Imaging Technology

A WSI scanner is a robotic microscope capable of digitizing an entire glass slide, using software to merge or stitch individually captured images into a composite digital image. Upon retrieval of the digital file, the captured image of a slide can be viewed, magnified and navigated spatially on a computer monitor in a similar way of an conventional light microscope (10).

In general, WSI consists of two processes: (1) digitalization of glass slides using specialized hardware (scanner composed of an optical microscope and digital camera connected to a computer), which generates a digital image and (2) digital image viewing and/or analysis through a software responsible for image creation and management (10). Each virtual image represents an entire glass slide and the images acquired through this technology are often referred to as a whole slide images, WSIs, whole slide scans or digital slides (12).

WSI technology has consistently developed during the last decade and currently there are several commercially available scanners capable to acquire digital images (12, 13). There are two main approaches to produce digital images. Most models use a tiling system, in which the original slide is acquired as tiles while others employ a line-scanning system that creates linear scans of tissue areas (12). Both methods require the tiles or line scans to be stitched together and smoothened by specialized software to create a single digital image of the histologic section. In addition to the scanning strategy, digital slide scanners vary in some of their features and capabilities, such as scanning capacity (100–200 slides in a single batch), objective availability (20x or 40x objectives) and image resolution (0.25–0.5 μm per pixel) (10). Most scanned images for histopathological analyses are acquired at bright field light microscopy with the 20x objective which is considered the standard magnification for digital slides and appropriate for most analyses (10). Some slide scanners can also be equipped with oil immersion lenses or fluorescent scanning capabilities (10).

Technological advances now allow WSI to be relatively fast and the images have high resolution. For example, to facilitate automation, new generations of slide scanners have incorporated tissue identification abilities, allowing the scanner to localize the tissue on the slide, and/or auto-focusing methods (14). Moreover, WSI has proved particularly suitable for applying deep learning algorithms that can assist human analysis of digital images of tissues (10, 15). These algorithms are able to extract all relevant parameters from WSI scans in a fully automated fashion by comparing tissue sections or even single pixel colors to previously defined categories (15). WSI combined with deep learning algorithms can perform histopathological assessment of large tissue sections in a significantly more time-efficient way and it will make tissue evaluation both more sensitive and more specific (15).

## WSI for Clinical Practice of Liver Pathology

WSI offers a convenient platform for measuring histopathological features of the liver and other pathological specimens, but its implementation in the clinical practice is still in progress (16–20). Validation of WSI is crucial to ensure that diagnostic performance based on digitized slides is at least equivalent to that of glass slides analyzed under conventional light microscopy, as established by the College of American Pathologists—CAP (21).

A comprehensive validation study using 176 needle liver biopsy specimens associated with different types of diseases such as autoimmune hepatitis, primary biliary cirrhosis, toxic acute hepatitis, acute and chronic hepatitis B, chronic hepatitis C, metastatic carcinomas, hepatocellular carcinomas, cystic fibrosis, and schistosomiasis showed a high intra-observer concordance between the diagnoses obtained with analyses through WSI (without application of algorithms) and conventional light microscopy (17). These authors concluded that WSI is safe for histological diagnosis of liver biopsies, including native and transplantation specimens (17). More recently, a pivotal study of 1992 biopsy samples from 20 organ systems, including 49 samples of neoplastic liver/bile duct compared WSI with conventional light microscopy for primary diagnostic in surgical pathology (18). Following the guidelines of the CAP for validation studies of WSI, including “diagnostic concordance between digital and glass slides for the same observer,” it was demonstrated that WSI is non-inferior to conventional microscopy “for the purpose of making a primary diagnostic in surgical pathology” (18). In a recent review, it is also highlighted that, in parallel to increased incorporation of WSI into diagnostic practice, it is important to validate the viewing systems as well as the security systems for remote locations (20).

While few validation studies have compared WSI and conventional light microscopy in the field of liver pathology, diverse technical approaches associated with WSI, including new algorithms, have been described for quantification of histopathological aspects of liver disorders such as cell alterations in hepatocellular carcinoma (HCC) and detection of hepatic fibrosis and steatosis. These studies are discussed in detail below.

## WSI in Hepatocellular Carcinoma Diagnosis

Hepatocellular carcinoma (HCC) comprises 75–85% of primary liver cancer cases, which are one of the leading causes of mortality by cancer in the world (22). Hepatocarcinogenesis usually occurs following previous liver damage, frequently related to hepatitis B and C, cirrhosis and some hereditary diseases (23, 24). Histologically, HCC is characterized by highly variable morphologies, which range from a pseudo-glandular or acinar growth pattern to a more compact semblance or even looking similar to normal liver tissue (25). HCCs are mostly classified using the Edmondson-Steiner grading system (26), which categorizes HCC into four stages, from a most differentiated (G1) to a least differentiated state (G4), where higher grades indicate greater malignancy (27). This classification is used for directing the appropriate treatment, mainly in early stages when there are better chances for a good prognosis (25).

**Figure 1:**
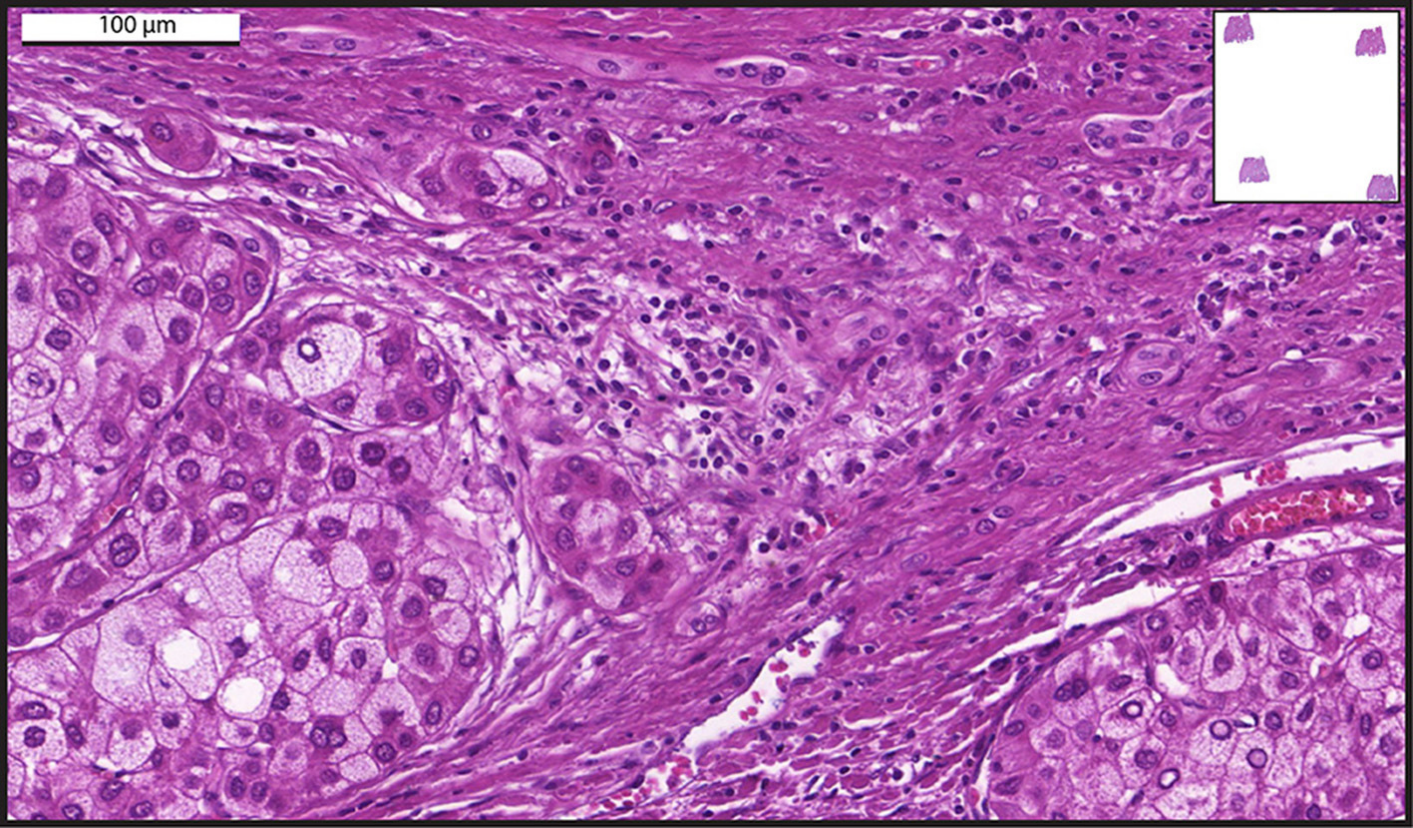
A representative whole-slide image of a human liver biopsy showing hepatocellular carcinoma. Sample was stained with hematoxylin and eosin and viewed with a digital scanner.

The introduction of WSI to acquire images from HCC (Figure 1) has motivated different groups to design algorithms for automatically grading HCC biopsies (28–32). Whole-slide images have been analyzed by software that recognizes differences and patterns in the image pixels indicative of specific HCC cell alterations in the nuclear area, circularity, and texture of hepatocytes; nuclear-cytoplasmic ratio and/or hepatic trabecular thickness. These analyses enabled HCC specimen grading with high accuracy (28–30, 33). Similar computational analyses from whole slide images obtained from liver biopsy specimens were also used to distinguish early well-differentiated HCC from non-cancerous hepatic tissues with a consistent classification (34). In summary, in the field of tumor pathology, advances in image processing and statistical methods have allowed greater refinement in the design of algorithms with an elevated potential in terms of diagnosis (35, 36).

## WSI in Liver Transplantation

Another field where the use of WSI as a histological assessment tool is emerging is that of transplantation medicine. In the US, the liver is the second most transplanted organ (https://unos.org/transplant/), behind only the kidney. Liver transplantation is used for a wide range of hepatic pathologies, most notably but not exclusively, infection (37), alcohol-related cirrhosis (38, 39), and hepatocellular carcinoma (40). Histologic assessment is one of the core aspects of transplantation medicine, both pre- and post-transplantation. In the pre-transplant phase, a histopathologist is responsible for assessment of biopsies of both the patient's liver, to confirm the diagnosis and therefore the need for transplantation, as well as the donor's liver, where it is of crucial importance that this is indeed a healthy liver. In the post-transplantation phase, monitoring of the allograft inside the patient is vital for continued healthy functioning of the liver graft in the patient, as a number of post-transplantation complications can arise, including infection of the graft or rejection of the host to the graft which should be detected promptly. For all aspects of the assessment, conventional light microscopy remains the golden standard (41), although, as discussed before, inter-observer variability have shown to be suboptimal in this process due to the challenging nature of the assessment (42).

Several studies have been conducted to investigate the additional value of WSI over conventional light microscopy in the transplantation medicine setting. Recently, Girolami and colleagues have reviewed the current literature on this topic for both liver transplants, but also for other types of organ transplantation (42). The overall conclusion is that digital pathology (mainly WSI, especially recently, and otherwise highly related technologies) has a good correlation with conventional light microscopy but with a higher potential when it comes to inter-observer concordance (42). This is because digital images of whole tissue sections are more easily shared and annotated, facilitating constructive collaboration on tissue assessment (42).

As WSI technology improves over time, the expectation is that within a foreseeable amount of time, digital pathology will overtake traditional forms of pathology in the field of organ transplantation and will ultimately lead to better patient-care (42). Also in the histopathologic assessment of allograft biopsies during monitoring, digital whole-slide approaches toward imaging open up possibilities of computational assessment of large tissue sections. This can complement classical histologic assessment by a pathologist in the short-term (43). In the long term, this can enhance the amount and pace of reliable information extraction from biopsies, benefiting both clinicians and patients by increasing the quality and completeness of the assessment (43). Perspectives and future directions in this regard are expertly reviewed by Wood-Trageser and colleagues.

## Liver Fibrosis Assessment with WSI

Liver fibrosis is a common histological consequence of the wound-healing process connected with chronic liver diseases, such as viral and helminthic infections; metabolic-, biliary- and autoimmune disorders (44–47). It is closely related with portal hypertension, which is a hemodynamic complication and one of the leading causes of mortality (48, 49).

Histological analysis of liver biopsy is considered the golden standard for determining the level of liver fibrosis (44, 49–52). Assessment and staging of liver fibrosis is, thus, of crucial importance for diagnosis, prognosis, preoperative indicative examination and therapy effectiveness monitoring (3, 44, 47, 51, 53). However, whereas classical light microscopy techniques are currently the daily clinical practice for liver fibrosis evaluation, histological quantitative analysis is considered subjective and dependent on the experience of the pathologist and the quality of the samples (54). Overall, there is not a single universally accepted system for staging fibrosis (54). Therefore, more objective and improved quantitative methods are desired (49, 54).

Digital image analysis was put forward as a potential solution and, in the late 1990s, experiments started in which fibrosis would be assessed and monitored in hepatitis C-affected patients using this novel technique (55). Further development of computational technology led to the conception of more advanced methods for extracting parameters from digital images. The developers claimed that these methods would automatically and accurately evaluate fibrosis in a tissue (56). Moreover, computer assisted-digital image analysis showed significantly less intra- and inter-observer variability (56, 57).

**Figure 2:**
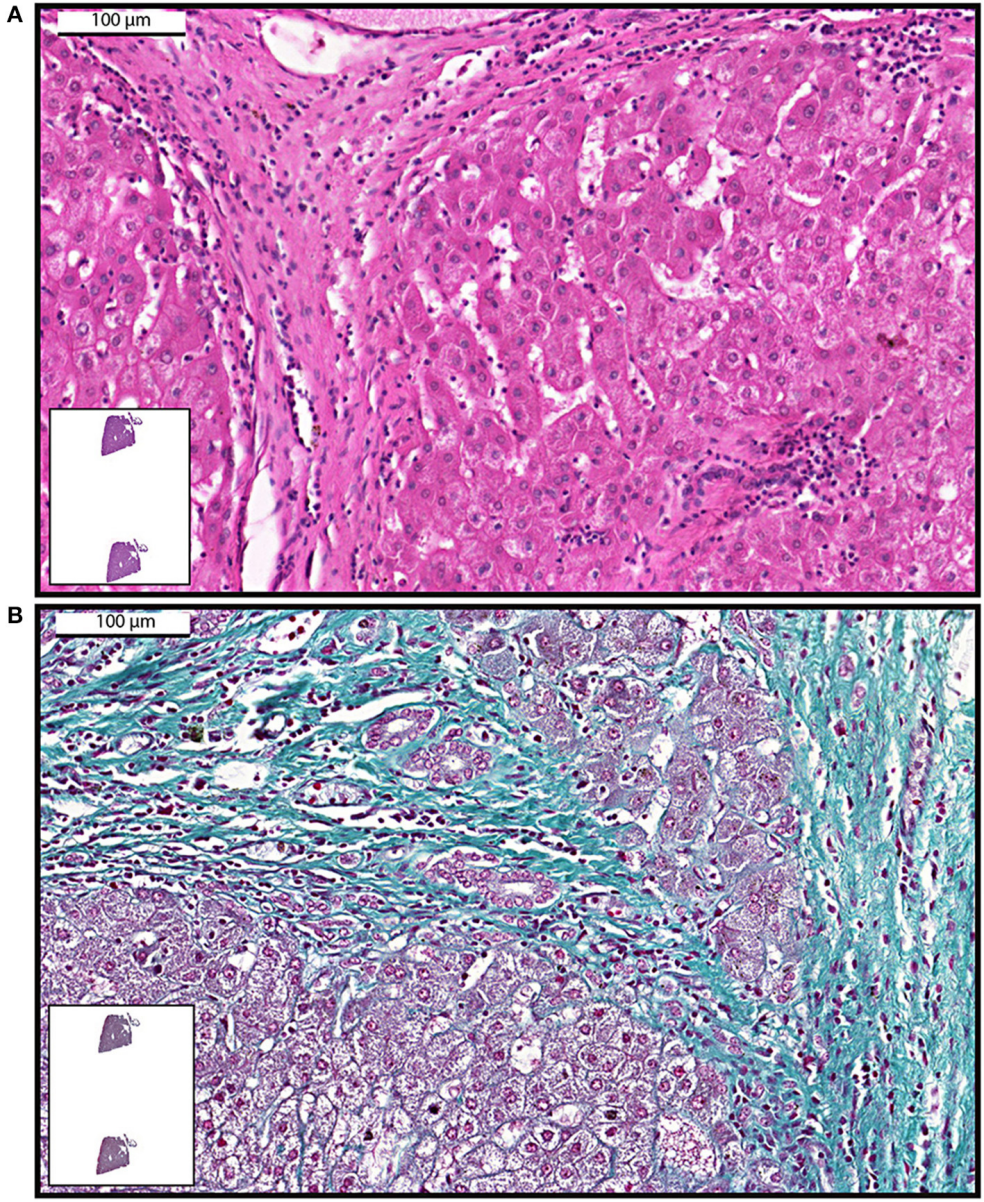
Examples of whole-slide images of human liver biopsies showing fibrosis. Samples were stained with hematoxylin and eosin **(A)** or Gomori trichrome **(B)** and viewed with a digital scanner.

WSI emerges as an advanced method for acquiring digital images for histopathological analyses of liver fibrosis (3, 6, 52, 54, 58, 59). Examples of images acquired with this technology demonstrating liver fibrosis are shown in Figure 2. WSI combined with computational methods was applied to liver biopsies specimens from chronic viral hepatitis (6, 59) and non-alcoholic fatty liver disease (NAFLD) patients (3) to monitor disease progression by quantification of both collagen and elastic fibers. Hepatic fiber amounts after staining with routine stains (for example Elastic van Gieson) were then quantitated in acquired whole-slide images based on a pixel-by-pixel evaluation of the ratios of the areas of collagen and elastin fibers to the total area of the biopsy samples (3, 6, 59). The data showed that morphometric analysis of the total amount of fibrosis in a biopsy is a more sensitive measure to determine fibrosis levels over the course of disease progression as compared with classical histological staging (6). High-resolution whole-slide images enabled precise color classification, with accurate detection of even fine connective tissue fibers (6). It is also highlighted that WSI seems indispensable to quantify structures occupying small amounts of space in clinical specimens of the liver, such as elastin (59, 60). Because accumulation of elastic fibers in addition of collagen fibers plays a role in advanced fibrosis, the detection of elastin in the liver using computational analysis is considered important and can be associated even with the development of HCC (59).

WSI plus computer assisted-digital image analysis showed to be a reliable approach to study liver fibrosis by uncovering a non-linear relationship between fibrosis stage and fiber amount in liver biopsy specimens from patients with NAFLD (3). The authors claimed that upon validation, these techniques might provide a better understanding of NAFLD progression with the improvement of management procedures for patients with NAFLD (3). On the other hand, amidst all the promising reports on WSI for liver fibrosis assessment, Jedrzkiewicz and colleagues provided a critical note. According to their research, the total quantity of fibrosis, as usually evaluated with WSI in this context, shows little diagnostic value when low levels of fibrosis are present (54). Rather, qualitative patterns of fiber deposition are more important at this stage of fibrosis.

In conclusion, WSI applied to the study of liver fibrosis show great promise as a means to quantify fibrosis more objectively (54). However, the diagnostic value seems not necessarily superior to the current classical microscopy approaches at lower levels of fibrosis (54). Improvements in qualitative assessment can be effectuated in future analysis software development.

## Hepatic Steatosis Evaluation Using WSI

Hepatic steatosis (fatty liver) encompasses a wide spectrum of conditions characterized by intracellular accumulation of lipids and subsequent formation of lipid droplets in the cytoplasm of hepatocytes (1, 61). Hepatic steatosis is associated with both alcoholic and NAFLD, which comprises varied diseases spanning from non-inflammatory isolated steatosis to steatohepatitis, hepatic fibrosis and cirrhosis (62, 63). In the last decade, NAFLD has received considerable attention, as it is one of the major complications of obesity, which, in conjunction with metabolic syndrome, has been considered a global epidemic (63). Progression of NAFLD may also lead to HCC (63).

The appropriate evaluation of disease severity, that is, to confirm or exclude steatohepatitis in a patient with chronically-increased liver enzymes and image-detected steatosis, requires histological examination (1). Although a search for non-invasive diagnostic approaches has been pursued and the use of liver biopsy restricted, this procedure is still considered the gold standard for determining the distribution and quantification of fat in the liver (1, 2). In human biopsies, steatosis grade evaluation is important to establish and monitor the evolution of diseases in which lipid accumulation is present (64), predict the suitability of liver grafts for transplantation and the risk of hepatic resection (65) and can also be used as a risk indicator for HCC. However, routine steatosis quantification in clinical practice usually involves a lengthy and subjective visual interpretation of the histological slides through a microscope (66). Grading systems to score steatosis are subject to inter-observer and intra-observer variability (67).

As noted for liver fibrosis, technological improvements in slide scanning, digital image analysis, and development of algorithms have been allowing a more objective and comprehensive evaluation of hepatic steatosis in human samples (68–71). Roy and colleagues propose a more precise image analysis method to recognize, quantify steatosis areas in whole-slide liver tissue microscopy images (70). This method consists of whole tissue component extraction, steatosis detection, and segregation of the overlapped steatosis component. More recently, a quantification algorithm was developed to detect potential steatotic hepatocytes in whole slide images and distinguish these images from similar objects, such as blood vessels, bile ducts, and tissue tearing (71).

## WSI for Research in Liver Pathology and Biology

### Applications to Research Pathology

While the application of WSI to the clinical practice is still ongoing, this technique has been proved of great value for research purposes in experimental animal models of liver disease. Several studies have been conducted in animal models of hepatic steatosis with application of WSI (72–76). For example, WSI and digital image analysis have enabled zonated quantification of steatosis with identification of differential steatosis patterns in mice submitted to dietary-induced steatosis and thus with potential application to help diagnosis, especially in the context of subtle changes (75). A more recent work used concepts of tile-based hotspot analysis (focused scores) applied to whole slide images in order to refine the quantification of small differences in steatosis in histological images obtained from rodents as models with potential use in research studies, for example to reduce the number of samples needed for demonstrating significant effects (76).

In other applications for research purpose, our group used WSI to study different aspects of the liver from naturally and experimentally animal models infected with the parasite *Schistosoma mansoni* (77–79). This organ is one of the main targets of schistosomiasis mansoni, a neglected tropical disease that causes marked chronic morbidity in humans with development of a severe granulomatous reaction against the parasite eggs trapped in the hepatic tissue (80–82). The size, number, distribution and the evolutional stage of liver granulomas are morphological parameters that need to be carefully investigated to understand diseases from varied etiologies and to help to arrive at an accurate diagnosis (83, 84). This means that a qualitative and quantitative evaluation of granulomas is required to achieve a reliable depiction of the disease. Moreover, although granulomas are structures readily observed in conventional microscopic images, they greatly differ in terms of size and distribution throughout the tissue, thus needing analysis of large tissue areas.

**Figure 3:**
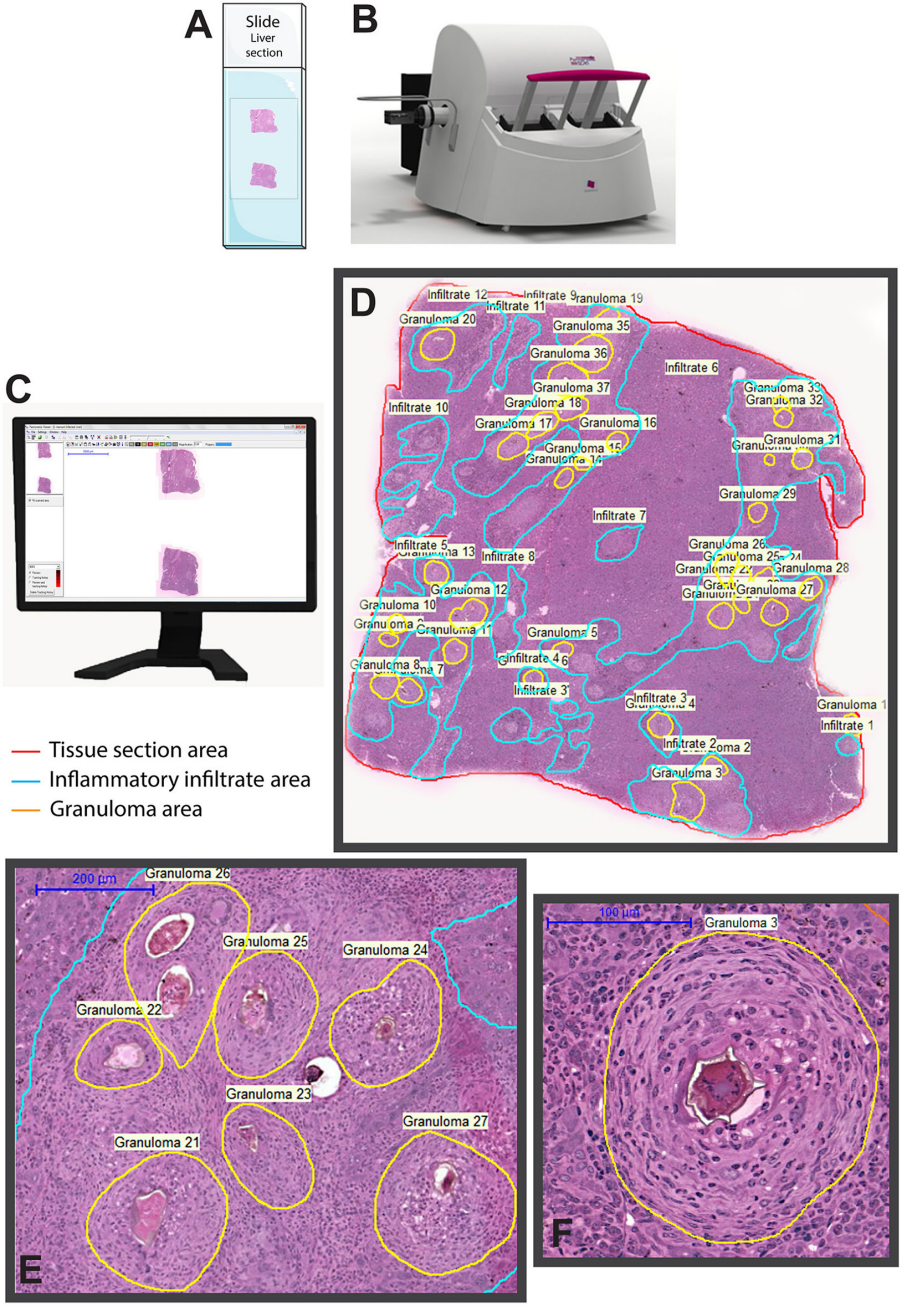
Whole slide imaging of a liver section showing granulomas elicited by *Schistosoma mansoni* infection in mice. After loading the slides **(A)** in the scanner **(B)**, a built-in digital camera captures the entire tissue section and viewer software generates a high-resolution digital slide **(C)**, which can be assessed by the operator by selecting and marking the area (s) of interest for qualitative and quantitative analyses. In **(D)**, a representative digital slide in which granulomas and non-granulomatous inflammatory regions were manually outlined for subsequent morphometric evaluations using associated software. In **(E,F)**, granulomas are seen at high magnification. The equipment and software illustrated in this figure are *3D Histech Pannoramic* scanner and *Pannoramic Viewer 1.15.2 SP2 RTM* software, respectively.

We provided the first detailed WSI characterization of hepatic granulomas with multiple quantitative evaluations using associated software (Figure 3): (i) Identification of types of granulomas according to their evolutional phase; (ii) Frequency of each granuloma stage; (iii) Total area of hepatic tissue taken by granulomas, that is, measurement of the total tissue area related to the granulomatous response; (iv) Tissue area taken by inflammatory infiltrates, that is, quantification of the area occupied by accumulated leukocytes in the hepatic tissue outside the granuloma; and (v) number and proportion of immune cells within granulomas (78). Moreover, since granulomas are dynamic structures composed of several cell populations and WSI has the advantage to show single-cell details, WSI analyses also allowed excellent identification and scoring of inflammatory cells such as eosinophils within schistosomal granulomas during both acute and chronic experimental infections (78).

**Figure 4:**
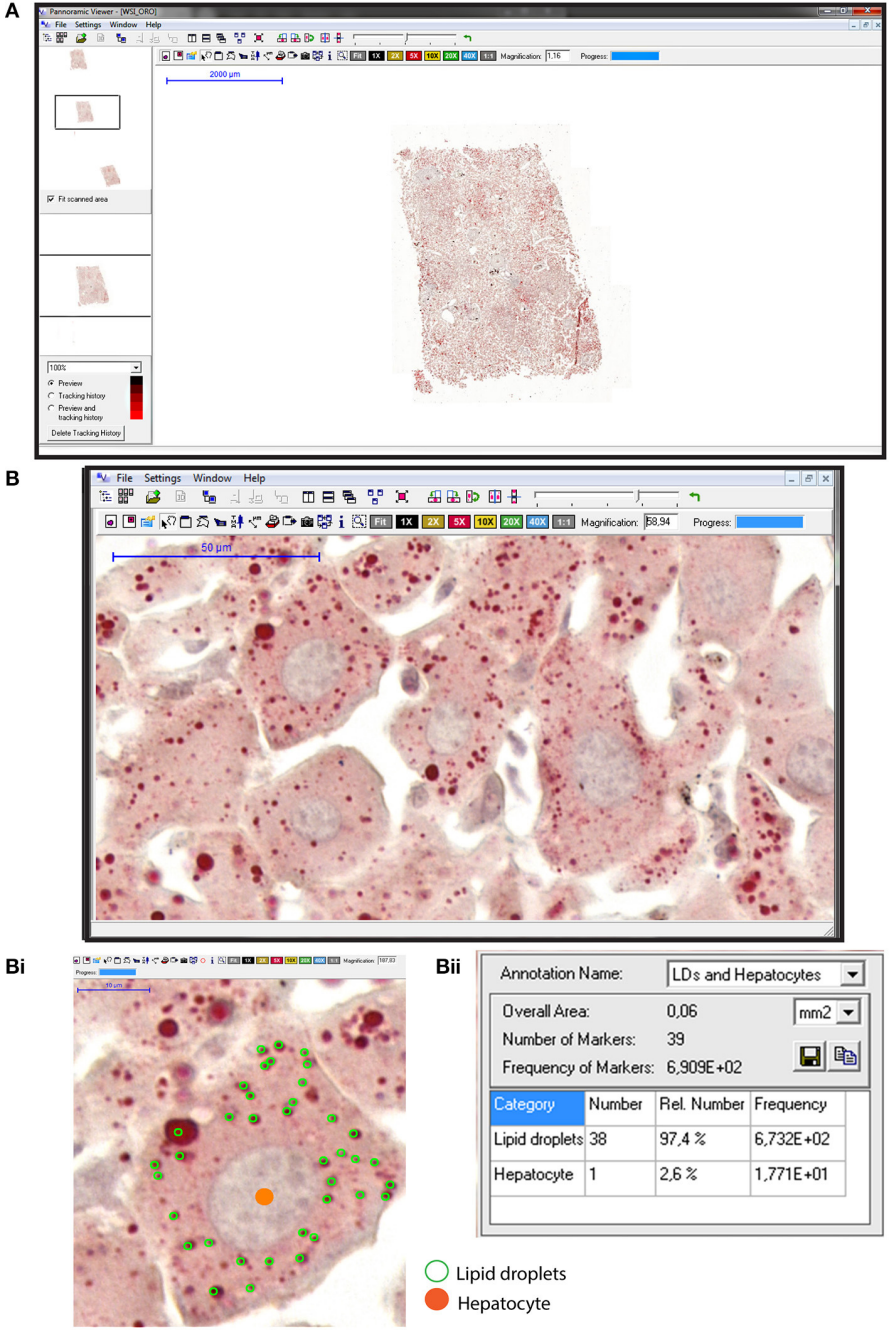
Hepatic steatosis evaluation using whole slide imaging. **(A)** A representative view of an entire liver section stained with Oil Red O (ORO) and counterstained with hematoxylin-eosin (HE). Observe at high magnification in **(B)** a tissue area with numerous lipid droplets (LDs) seen as round structures stained in red within hepatocytes. In **(Bi)**, hepatocyte nuclei were marked in orange whereas cytoplasmic LDs were outlined in green within individual hepatocytes. Quantification of the marked elements **(Bii)** with associated software enables steatosis evaluation in large areas of the liver. ORO and HE staining were performed on cryosections from rat liver fragments fixed in buffered paraformaldehyde. After staining, glass slides were digitized using an automated scanner.

Our studies applying WSI to study large areas of the liver from experimentally infected animals also enabled to capture the development of an intriguing “beneficial” steatosis during the natural schistosomiasis mansoni infection in wild reservoirs (77). By applying WSI and image analysis to liver sections stained with oil red O (ORO), a histochemical staining for lipids (85), we detected a significant increase of the lipid droplets numbers not only within individual hepatocytes (Figure 4) but also in terms of hepatic tissue area in infected animals compared to uninfected controls (77). Accumulation of lipid droplets in the infected liver during the natural *S. mansoni* infection occurs without affecting the liver functional activity and co-exists with a low incidence of inflammation, likely acting as a protective mechanism for dealing with the infection (77).

Altogether, WSI has proved to be powerful in providing an accurate and whole view of several pathological aspects of the liver in the context of research with implication to better understanding of liver diseases.

### WSI to Understand Liver Biology

Since WSI generates high-resolution images and offers the possibility to perform high throughput of cellular analyses in the context of whole tissue, it has also been used to extract more biologically significant cellular information from the healthy liver tissue. For example, WSI and automated image analysis were used to study liver epithelial diversity in human tissue sections immunolabeled for hepatic progenitors cells (4). This approach enabled identification and quantification of preexistent complex cell phenotypes, that is, hybrid cells showing phenotypes between hepatocytes and biliary epithelial cells, the two main epithelial cell types classically described by routine histology (4). The authors reported a vast increase in data acquisition on cell numbers/types and nuclear/cytoplasmic ratios as well as the location of these hybrid transitional cells compared with classical microscopy approaches (4). The demonstration of such significant cell diversity in the healthy liver is important to understand how the hepatic cell populations proliferate, differentiate and respond to injury. In fact, the liver has a unique capability of regeneration from various injuries and WSI associated with quantitative image analysis has been helping to understand its regenerative competence (86). Another comprehensive study using this technique identified hybrid periportal hepatocytes as highly efficient cells acting in the repair of the liver (87). By using WSI to locate these cells and their responses to liver damage, the authors demonstrated that, despite their readiness to proliferate on a large scale, hybrid periportal hepatocytes showed little tendency to develop into a cancerous growth (87). This is a counterargument to the dogma that cells that show high proliferation rates universally have an increased risk of turning cancerous. The fact that hybrid periportal hepatocytes are highly efficient during liver regeneration, but not associated with cancer risk, makes these cells of potential use in the treatment of diseases affecting the healthy functioning of the liver (87).

A three-dimensional (3D) model of sectioned tissue provides great insight into structural features and spatial relations that can be very relevant when understanding health and disease properties of an organ (88). Up until now, production of such 3D models has been technically challenging, since traditional microscopes can only capture small parts of a section at once. WSI is able to transcend this limited field of vision and provide scans of the whole tissue section, as previously discussed. This makes WSI a highly effective way of mapping and imaging large tissue sections at once that can later be used to reconstruct a 3D model (88).

Several authors have been exploring the large-scale visualization provided by WSI for high-resolution reconstruction of the liver in a 3D fashion with the purpose to better understand the structure of this organ (88–90). One important aspect investigated was the mapping of the hepatic 3D vasculature (89, 90). These authors presented frameworks for 3D vessel structure analysis on whole slide images of liver tissue sections, with effective qualitative and quantitative results. These models are important to understand the massive and complex organ vasculature, which is puzzling to study when cross-sections from different image slides are evaluated (89, 90).

The understanding of the liver vasculature has implications in the field of liver regeneration, as hemodynamic changes shortly following liver tissue damage are largely unexplored but are thought to be highly important in initiating the regenerative response. Moreover, WSI can be used to visualize morphological changes in the tissue along a blood vessel during the phases of increased shear stress, which are thought to play a large role in regeneration (91). Thus, the major advantages of WSI for these applications is that it allows the tracking of vessels through large sections of the tissue, including branches, while also taking into account the surrounding tissue.

## Limitations

Overall, WSI has been successfully applied to the understanding of liver disorders/biology, but limitations exist, especially for routine clinical practice. Most general limitations of WSI are also applied to the field of liver pathology since they can affect data collection/interpretation of liver biopsies. For example, considering that the quality of digital slides depends on the quality of the original slide, the first concern is related to pre-imaging steps, such as tissue collection, handling, fixation, processing/embedding, sectioning, and staining/labeling (10).

While the quality of the digital slides is important to achieve the best possible diagnostic accuracy in the clinical practice of liver pathology, in experimental studies, each of these pre-analytical variables must be clearly defined to make accurate comparisons between study groups. Fixation can have an impact on both morphology and the ability to detect antigens and therefore the choice of tissue specific methods of fixation should be considered (92). Likewise, uneven tissue staining can lead to variations in pattern recognition of image analysis and in the software's ability to segment tissue sections (10). The lack of standardization of these steps can produce significant variability between tissue sections thus influencing automated measurements of histopathological parameters (10, 93).

To enable color consistency during WSI analyses, some strategies have been effectively applied, such as the use of color calibration slides (93, 94) and automated algorithms (95). By using WSI from hematoxylin and eosin-stained histological slides of rat liver with different amounts of confluent necrosis, Bejnordi et al. proposed a novel algorithm for standardization of whole-slide histopathological images (95). In fact, standardization of the colors shown by digital slides has been considered a critical aspect of digital pathology implementation (93–95).

Specimen thickness can also be an issue to acquire adequate digital images with digital scanners. The overall thickness of specimen can influence the color appearance of stained tissue section (93) and seems to be a more relevant issue for immunohistochemical preparations analyzed by WSI. The recommendations are, when scanning single-plane images, standard tissue thickness (4–7 μm) will give the best results, that is, better digital image quality enabling consistent analysis using WSI (93, 96). Optimal results seem to be achieved with 4 μm (96, 97), while very thick samples can compromise image analysis. For example, Isse and colleagues reported that automatic nucleus segmentation is still a challenge when cell nuclei overlap in thick sections (4, 96). Moreover, specimen thickness should be uniform, since tissue thickness variation influences z-plane focus (96). In fact, consistency in the thickness of tissue sections greatly facilitates the registration and alignment of each whole-slide image necessary for these functions (97). New scanner systems' technologies available more recently should solve the focus problem (14).

Other slide-related issues such as prior ink markings, extra or wet mounting media, plastic coverslips, or thick slide labels may compromise the quality of the digital images. Sections placed close to slide edges or small coverslips will affect quality and image analysis since these edges can be interpreted by software as tissue. Finally, the thickness of the glass slides itself can be a limiting factor that prevents a proper load of the slides in the digital scanner, thus inducing equipment malfunction. Very thick glass slides, as used in the past for pathological samples, are not adequate for digital scanning. Appropriate glass slide thickness should be in accordance with the digital scanner manufacturer's instructions. Therefore, high-quality digital imaging primarily requires high-quality specimen preparation, and quality control procedures should be in place to ensure the highest quality scanned images possible (98).

Another point is the fact that, when automated image analysis is performed, software interfaces to WSI are not platform-agnostic and therefore multiple tools still require specialty informatics technologies. Moreover, to process and store extremely large image files can be a limitation (99). Slide scanner manufacturers have not adopted a common format for image files. Not only are these files pretty large, but they are also separated in multiple files organized in so-called “pyramids” to distinguish different levels of magnification. Thus, storage and sharing can oftentimes become difficult (12).

For an automated analysis using whole-slide images, some regions of the tissue may be considered non-informative and the software can require small alterations depending on the sample used (10). In liver biopsies prepared for HCC evaluation, for example, muscle, fat, and fiber regions are non-informative (28). Excluding or reducing these areas from an automated analysis, when possible, should minimize errors. On the other hand, as noted, considering specifically liver samples, some structural elements such as elastic fibers, which can be present in small amounts in liver fibrosis, requires appropriate algorithms for accurately detect them.

Finally, the high costs of implementation, workflow incorporation, user interface, and pathologist's comfort are limitations for the incorporation of this technology for clinical diagnosis (21). Initiatives to develop a multisite pathology informatics platform incorporating WSI and integrating medical and scientific staff to support high-quality pathology have been made (100).

## Final Remarks

While the field of digital pathology is continuing to advance, its role in the clinical practice of pathology is still being defined. In general, there is increasing interest in using WSI for diagnostic purposes in pathology, including routine diagnosis of liver specimens (16–20).

The introduction of WSI has been improving the diagnosis of routine needle biopsy specimens since this technique can show many slides stained with different staining methods at the same time, which is very useful in pathology (17). For example, in studies of the liver, glass slides prepared with specific techniques to demonstrate liver fibrosis steatosis or specific proteins (immunohistochemistry) can be feasibly compared with slides stained with HE to visualize general morphology. Moreover, WSI offers the possibility of making annotations/measurements and the opportunity for sharing and teleconsultation, thus facilitating rapid second opinions and discussions between investigators and pathologists (8, 17). WSI has also been well-accepted for educational purposes during training pathology (20).

The recent growing of WSI application in liver pathology can be attributed to advances in image quality, improved technology of WSI scanners and software, increased computational power of computers, better network connectivity as well as relative ease of slide reproduction and distribution (17, 21). In basic research, WSI has been proved to be a powerful tool for a more refined view of liver biology (89, 90) and pre-clinical evaluation of liver responses to new compounds (101). In the clinical practice, the objective quantification of histological results is essential not only to define objective and well-established protocols for diagnosis, treatment, and assessment but also to ameliorate disease comprehension. However, automated image analysis of large quantities of data generated by WSI still requires more refined computational methods and additional studies to validate the routine diagnosis.

## Author Contributions

All authors contributed in part to writing and editing the manuscript and shaping the figures and approved the final version. RM prepared the final version of the manuscript and TS prepared the final figures.

### Conflict of Interest

The authors declare that the research was conducted in the absence of any commercial or financial relationships that could be construed as a potential conflict of interest.
